# Supplementary material for: Identification of photoperiod-induced specific miRNAs in the adrenal glands of Sunite sheep (Ovis aries)
Source: Front Vet Sci. 2022 Jul 22;9:888207. doi: 10.3389/fvets.2022.888207 (PMC9354845; doi:10.3389/fvets.2022.888207)
Supplement: Supplementary file 1 [file Data_Sheet_1.ZIP › Supplementary file/miRDeep2 file.zip!/miRDeep2 file/Know_Structure/LP42A1/oar-mir-30d.pdf]

miRBase precursor : oar-mir-30d
Total read count : 231753
oar-miR-30d read count : 230819
remaining reads : 934

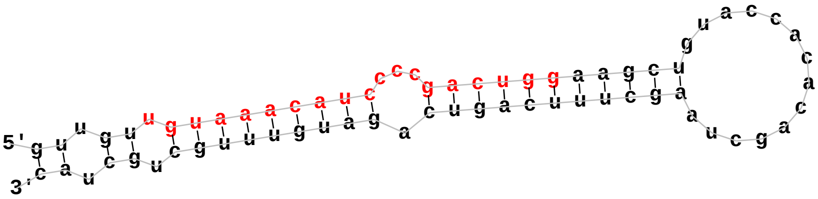

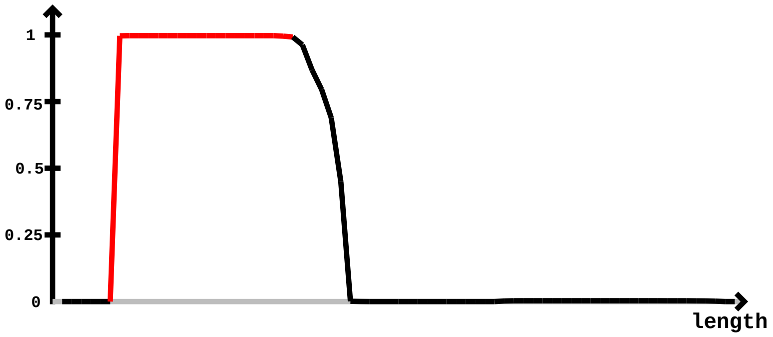

oar-miR-30d

```
5'- guuguuguaaacauccccgacuggaagcuguaccacacagcuaagcuuucagucagauguuugcugcuac   -3'    exp
    ...((.(((((((((...(((((((((((..............))))))))))).))))))))).))...    reads    mm    sample
    ....uuguaaacauccccgacuggaagcu.........................................    1        0     seq
    .....uguaaacaucccUgacu................................................    7        1     seq
    .....uguaaacaAccccgacu................................................    1        1     seq
    .....uguaaacauccccgacu................................................    390      0     seq
    .....uguaaacauccccgGcu................................................    3        1     seq
    .....uguaaacauccccgacug...............................................    777      0     seq
    .....uguaaacaAccccgacug...............................................    1        1     seq
    .....uguaaacauccccgacugg..............................................    6799     0     seq
    .....uguaaacauccccgacugga.............................................    21688    0     seq
    .....uguaaacauccccgacuggaa............................................    17030    0     seq
    .....uguaaacauccccgacuggaag...........................................    24522    0     seq
    .....uguaaacauccccgacuggaagc..........................................    55607    0     seq
    .....uguaaacauccccgacuggaagcu.........................................    103851   0     seq
    .....uguaaacauccccgacuggaagcug........................................    173      0     seq
    .....uguaaacauccccgacuggaagcugu.......................................    67       0     seq
    .....uguaaacauccccgacuggaagcugua......................................    6        0     seq
    .....uguaaacauccccgacuggaagcuguac.....................................    3        0     seq
    .....uguaaacauccccgacuggaagcuguaccaca.................................    1        0     seq
    ......guaaacauccccgacugg..............................................    3        0     seq
    ......guaaacauccccgacugga.............................................    8        0     seq
    ......guaaacauccccgacuggaa............................................    6        0     seq
    ......guaaacauccccgacuggaag...........................................    5        0     seq
    ......guaaacauccccgacuggaagc..........................................    25       0     seq
    ......guaaacauccccgacuggaagcu.........................................    87       0     seq
    .......uaaacauccccgacugga.............................................    1        0     seq
    .......uaaacauccccgacuggaagc..........................................    2        0     seq
    .......uaaacauccccgacuggaagcu.........................................    5        0     seq
    .........................................uaagcuuucagucagauguuu........    1        0     seq
    .............................................cuuucagucagauguuugc......    11       0     seq
    .............................................cuuucagucagauguuugcu.....    62       0     seq
    .............................................cuuucagucagauguuugcug....    42       0     seq
    .............................................cuuucagucagauguuugcugc...    140      0     seq
    .............................................cuuucagucagauguuugcugcu..    241      0     seq
    .............................................cuuucagucagauguuugcugcua.    4        0     seq
    ..............................................uuucagucagauguuugc......    3        0     seq
```

oar-miR-30d

```
guuguuguaaacauccccgacuggaagcuguaccacacagcuaagcuuucagucagauguuugcugcuac
.............................................uuucagucagauguuugcu.....    10    0    seq
.............................................uuucagucagauguuugcug....    8     0    seq
.............................................uuucagucagauguuugcugc...    32    0    seq
.............................................uuucagucagauguuugcugcu..    126   0    seq
.............................................uuucagucagauguuugcugcua.    3     0    seq
..............................................uucagucagauguuugcugcu..    1     0    seq
```
